# How dynamic adsorption controls surfactant-enhanced boiling

**DOI:** 10.1038/s41598-022-21313-1

**Published:** 2022-10-28

**Authors:** Mario R. Mata, Brandon Ortiz, Dhruv Luhar, Vesper Evereux, H. Jeremy Cho

**Affiliations:** Department of Mechanical Engineering, University of Nevada, Las Vegas, Las Vegas, NV 89154 USA

**Keywords:** Molecular self-assembly, Chemical engineering, Mechanical engineering, Colloids, Surfaces, interfaces and thin films, Surface assembly, Fluid dynamics

## Abstract

Improving boiling is challenging due to the unpredictable nature of bubbles. One way to enhance boiling is with surfactants, which alter the solid–liquid and liquid–vapor interfaces. The conventional wisdom established by previous studies suggests that heat transfer enhancement is optimized near the critical micelle concentration (CMC), which is an equilibrium property that depends on surfactant type. However, these studies only tested a limited number of surfactants over small concentration ranges. Here, we test a larger variety of nonionic and anionic surfactants over the widest concentration range and find that a universal, optimal concentration range exists, irrespective of CMC. To explain this, we show that surfactant-enhanced boiling is controlled by two competing phenomena: (1) the dynamic adsorption of surfactants to the interfaces and (2) the increase in liquid dynamic viscosity at very high surfactant concentrations. This dynamic adsorption is time-limited by the millisecond-lifetime of bubbles on the boiling surface—much shorter than the timescales required to see equilibrium behaviors such as CMC. At very high concentrations, increased viscosity inhibits rapid bubble growth, reducing heat transfer. We combine the effects of adsorption and viscosity through a simple proportionality, providing a succinct and useful understanding of this enhancement behavior for boiling applications.

## Introduction

Boiling is an extremely effective multiphase heat transfer process that transports vast amounts of energy through ebullition. Due to its effectiveness, boiling plays a vital role and is used in a wide variety of applications (e.g., electronics cooling^1^, compact heat exchangers and evaporators^2,3^, nuclear reactors^4^, etc.). Despite its ubiquity, we have a limited understanding^5–8^ of the complex bubble dynamics and other physical phenomena involved. We have an even more limited understanding of how to enhance this complex set of physical phenomena. The enhancement is quantified using the heat transfer coefficient (HTC), defined as

1 $${\text{HTC}} = q^{\prime\prime}/{ }\Delta T.$$

**Figure 1:**
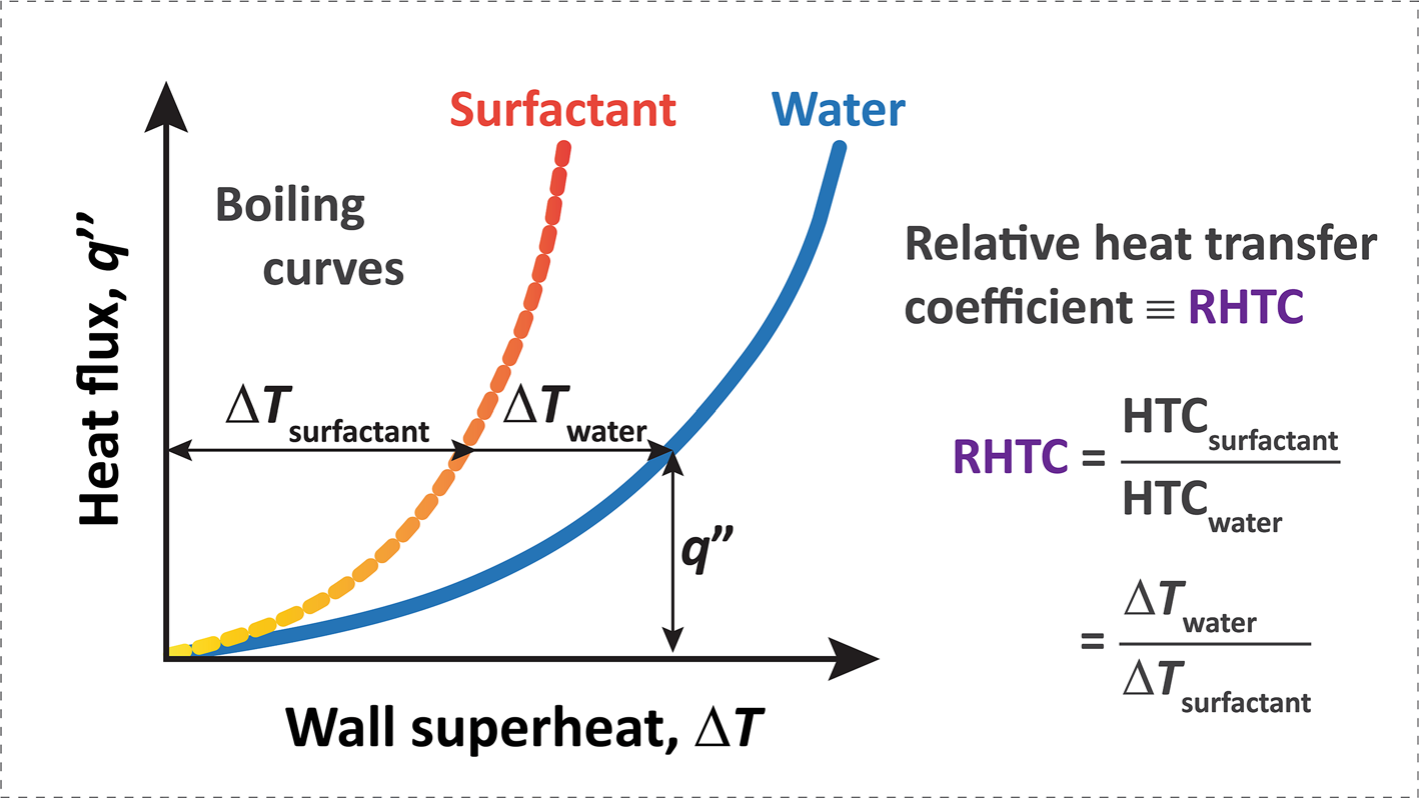
We quantify the degree of boiling enhancement with surfactants using a relative heat transfer coefficient (RHTC), defined as the ratio of HTC with surfactant over HTC of pure water at a given heat flux.

Here, $q^{\prime\prime}$ is the heat flux towards ebullition and $\Delta T$ is the wall superheat defined as the difference between heating surface temperature, $T_{{\text{s}}}$, and the saturation temperature, $T_{{{\text{sat}}}}$. Many approaches in recent decades have focused on incorporating surface micro and nano-sized features to enhance bubble nucleation and wetting of the surface to prevent dryout^9–16^. However, these extensive surface modifications can be expensive at large scales^16^. One potentially low-cost approach would be to add a small amount of chemical additive that modifies interfacial behavior: surfactants. Adding these surfactants in boiling water can sometimes enhance HTCs on the order of 10–1000%^17–19^. However, it should be noted that the definition of enhancement can vary from study to study, likely contributing to this wide range. Hence, in Fig. 1, we define a relative HTC (RHTC) to provide a consistent definition of enhancement in our study. In other cases, surfactants can degrade heat transfer, depending on surfactant type and concentration. Thus, there is a great need to study how much and what type of surfactants to use, requiring an understanding from a mechanistic and molecular perspective.

**Figure 2:**
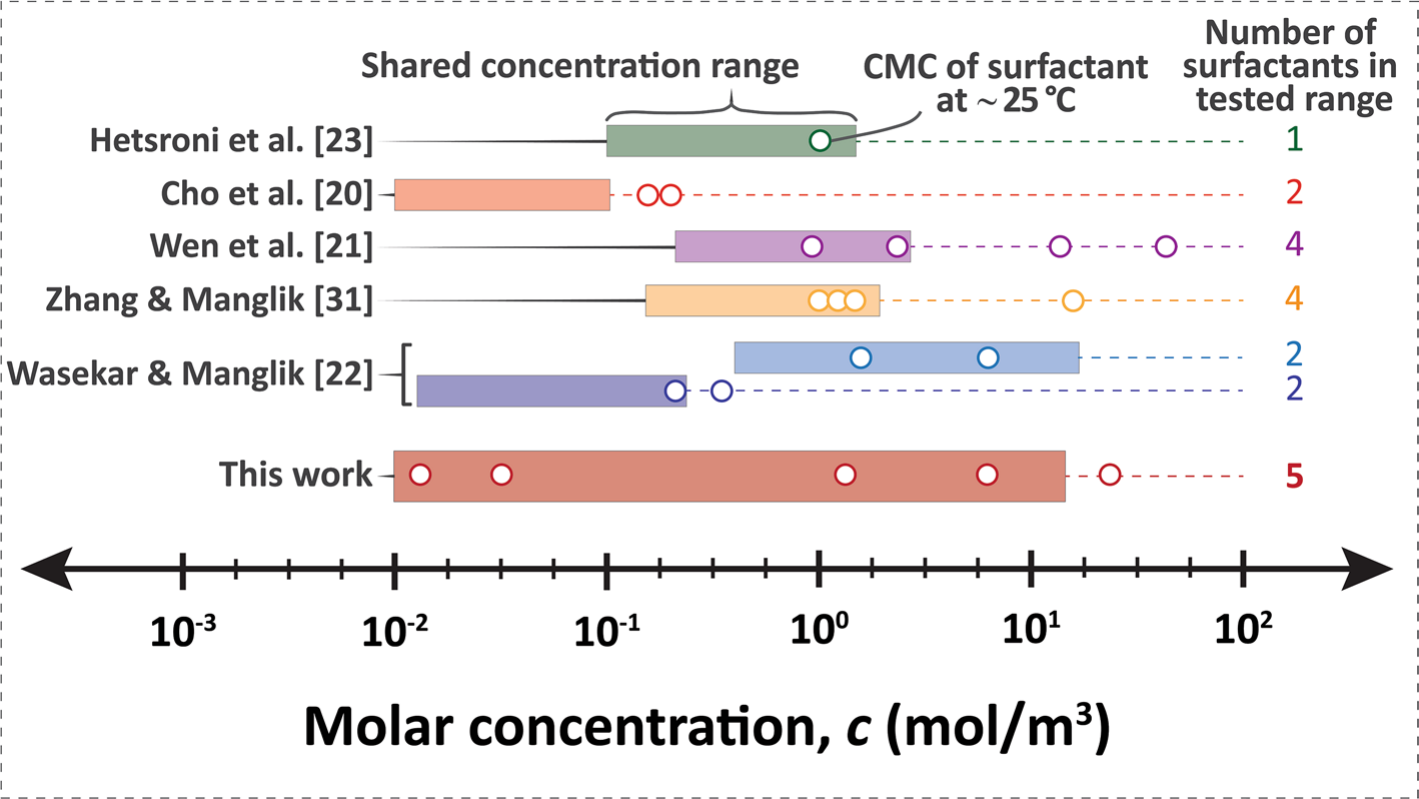
Our work encompasses more surfactants, a larger shared concentration range inclusive of all tested surfactants (rectangles), and a larger CMC range (circles) in comparison to previous studies.

Surfactants are amphiphilic molecules that once dissolved, adsorb to interfaces, and alter the interfacial properties of the liquid. It has been shown that generally increasing the concentration of surfactant in the boiling liquid provides more heat transfer at lower wall superheats, hence improving the HTC. However, adding too much surfactant has also shown to degrade HTC. This enhancement and degradation have been observed for nonionic, anionic, and cationic surfactants^17,20–28^, which suggests that surfactants behave similarly regardless of their ionic type. Thus, there is an open question about what the optimal concentration range is for a surfactant. Previous studies have concluded that this optimal concentration depends on the surfactant type and could coincide with the critical micelle concentration (CMC)^22,24,29–31^, a concentration where surfactants begin to aggregate into micellar structures. The CMC is a standard equilibrium property of surfactants that can be predicted^32^ or measured^33^ for many types of surfactants. Specifically, at concentrations above the CMC, if given enough time, an equilibrium concentration of micelles will form. However, we question the validity of an equilibrium description for a highly dynamic process like boiling. Depending on the surfactant type, the CMC can span many orders of magnitude from 10^–8^ to 10^2^ mol/m^3^. Despite this large range in CMC, most studies^17,20,23,24,29^ typically use surfactants (such as Sodium dodecyl sulfate, Triton X-305, Habon G, Cetyltrimethyl ammonium bromide, etc.) with CMCs in the range of 0.1–10 mol/m^3^. Furthermore, many existing studies only study a single surfactant^23,25,27,28,30,34–37^. Thus, we question the strength of the conclusion that the optimal concentration coincides with CMC if only a narrow range has been tested for very limited sets of surfactants. Furthermore, previous studies have only investigated concentrations close to its CMC value (within three orders of magnitude; Fig. 2)—no study has systematically looked at a large number of surfactant types over a broad range of concentrations.

**Figure 3:**
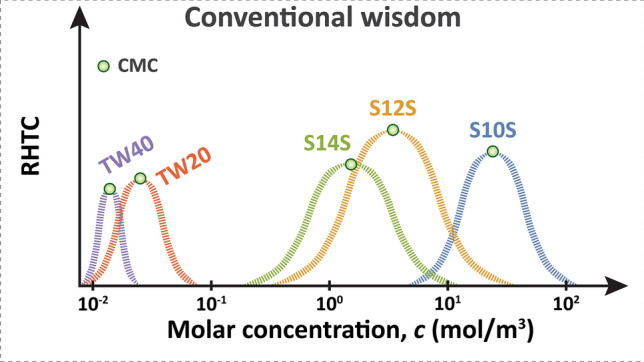
According to previous studies, the optimal boiling performance should be in concentration ranges near each surfactant’s respective CMC; thus, each surfactant should have its own separate peak in RHTC.

Based on the conclusions drawn from these limited concentration ranges (Fig. 2), we should expect to see separate optimal peaks of enhancements in HTC coinciding with each surfactant’s respective CMC (Fig. 3).

Our work reports the results of boiling heat transfer experiments using five different surfactants, namely, sodium decyl sulfate (S10S), sodium dodecyl sulfate (S12S), sodium tetradecyl sulfate (S14S), TWEEN 20 (TW20), and TWEEN 40 (TW40). The objective of this study is to test the conventional wisdom of separate optimal peaks in RHTC (Fig. 3) over a wide range of concentrations.

## Boiling results

The CMC of S10S, S12S, S14S, TW20, and TW40 surfactants ranges from 0.02 to 32 mol/m^3^, spanning four orders of magnitude, which is greater than previous studies (for full CMC values, see Table S.1, supplementary information (SI)). Furthermore, we adjusted the concentration from 0.01 to 14 mol/m^3^ (four orders of magnitude) for every single surfactant (Fig. 2), representing the largest concentration variation and diversity of surfactants compared to previous work. We performed over 150 pool boiling heat transfer experiments using a custom boiling chamber (details can be found in Figs. 10, S.1 (SI) and the Methods section; full boiling results in Fig. S.2, SI).

**Figure 4:**
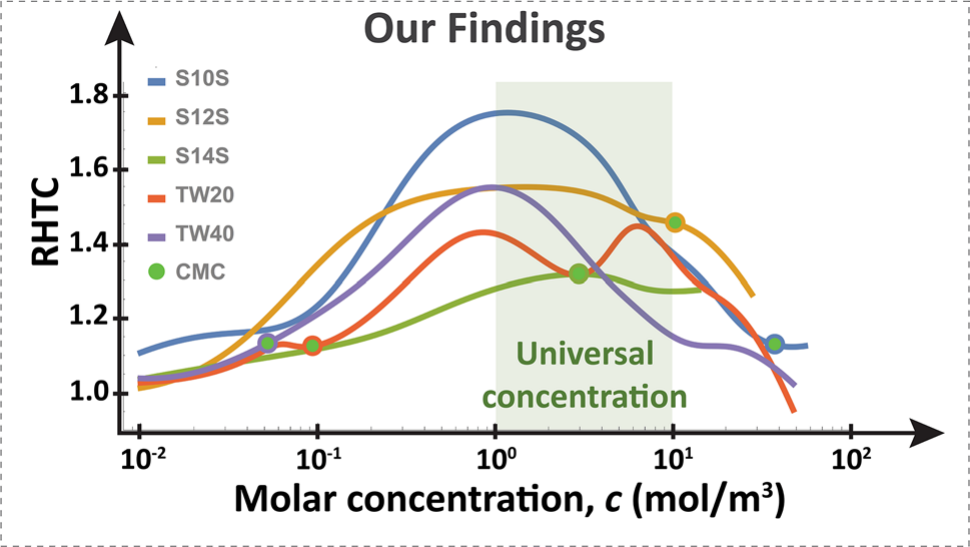
Our findings show that there is a universal, optimal concentration range of enhancement for a wide variety of surfactants, irrespective of their CMC. RHTC values are averaged in the range of 20–40 W/cm^2^.

Contrary to previous findings (Fig. 3), only a single universal peak in RHTC was measured (Fig. 4). That is, across surfactants of vastly different CMCs, they all responded with similar HTC enhancement, with a common, optimal concentration range around 1–10 mol/m^3^. This surprising result, completely counters conventional wisdom about equilibrium micellization behavior dictating boiling heat transfer enhancement. One would expect TW40 to have a peak in HTC near its low CMC value of 0.05 mol/m^3^, but we found that it peaks in the range of 1–10 mol/m^3^. Similarly, one would expect S10S, which has a CMC nearly 1000 × larger than TW40 to peak around 38 mol/m^3^; however, it also peaks in the range of 1–10 mol/m^3^. Thus, we found that there may be a universal concentration range of heat transfer enhancement that is independent of micellization and that something else must be mainly responsible for controlling this enhancement behavior.

### Theory

**Figure 5:**
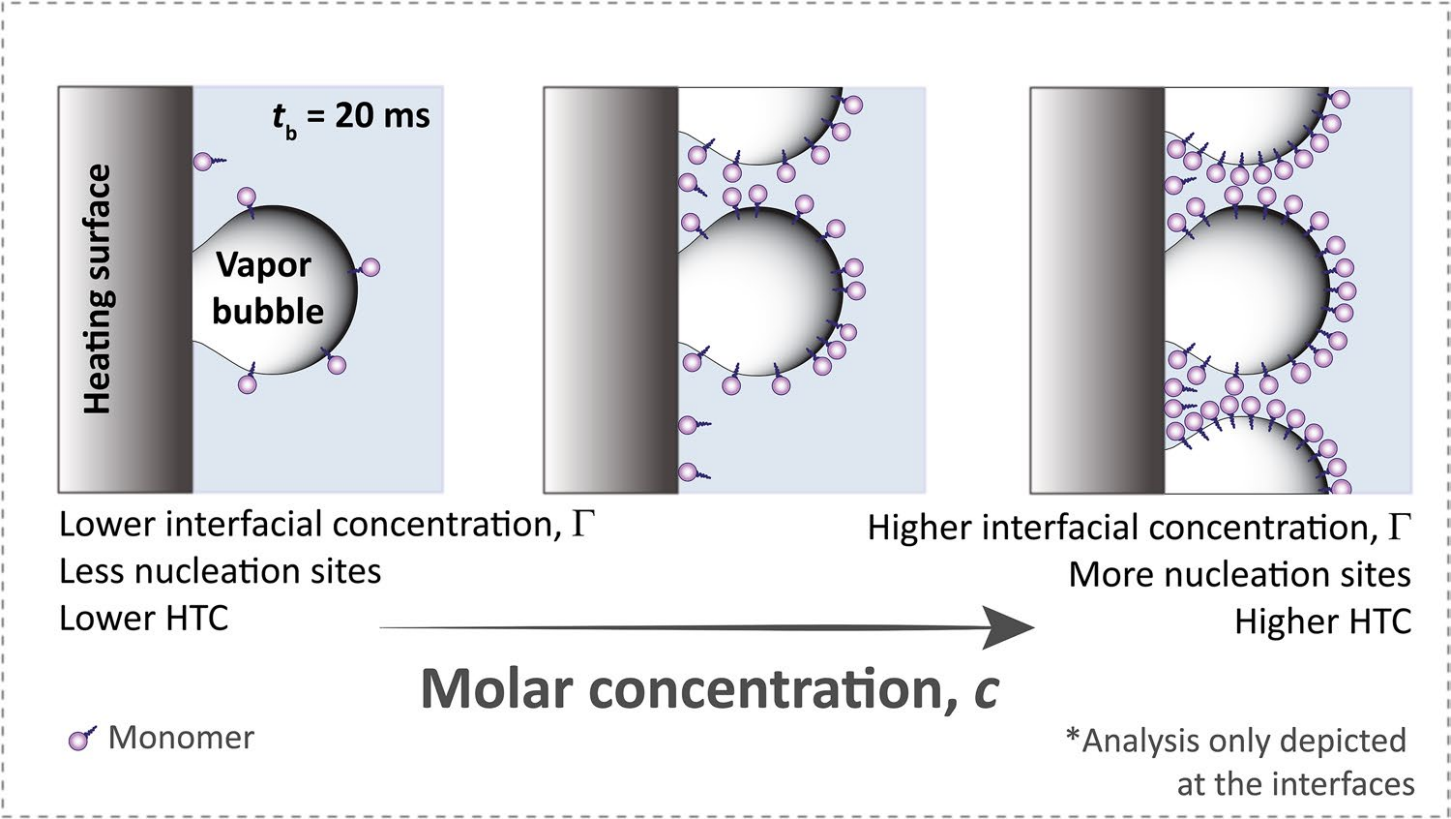
Dynamic adsorption of surfactants to the interfaces (solid–liquid and liquid–vapor) is responsible for HTC enhancement. With higher interfacial concentration, ${\Gamma }$, more bubble nucleation sites activate, resulting in higher HTC.

To understand this surprising universal behavior, we developed a dynamic description of boiling enhancement, as opposed to an equilibrium description based on the CMC. Motivated by the fact that generally having more surfactants at the interfaces (Fig. 5) enhances HTC as we tested previously^38^, we believe that HTC enhancement is set by how many surfactants can adsorb within a limited time window—dynamic adsorption. This time window corresponds to a bubble lifetime on the surface, $t_{{\text{b}}}$, estimated using Cole’s work^39^ to be approximately 20 ms (see Theory S.1, SI) for the boiling conditions encountered in our experiments. During this time window, surfactants diffuse from the bulk solution to the interface. This time-dependent diffusion behavior is true of any surfactant regardless of ionic type^40^.

As with any diffusion problem, the characteristic diffusion timescale, $t_{{{\text{diff}}}}$, is a lengthscale squared, $h^{2}$, divided by a diffusion coefficient, $D$. According to Ferri & Stebe^40^, this lengthscale is $h = {\Gamma }_{{{\text{eq}}}} /c$ where $c$ is the molar concentration and ${\Gamma }_{{{\text{eq}}}}$ is the equilibrium surface concentration determined by the Langmuir isotherm: ${\Gamma }_{{{\text{eq}}}} = {\Gamma }_{{{\text{max}}}} \frac{{K_{{\text{L}}} c}}{{1 + K_{{\text{L}}} c}}$ where ${\Gamma }_{{{\text{max}}}}$ is the maximum surface concentration where every possible adsorption site is filled (related to the inverse of the cross-sectional area of the surfactant molecule), $K_{{\text{L}}}$ is the Langmuir equilibrium adsorption constant. Thus, the characteristic timescale is

2 $$t_{{{\text{diff}}}} = \frac{{h^{2} }}{D} = \frac{{{\Gamma }_{{{\text{max}}}}^{2} }}{{D{ }c^{2} }}\frac{{K_{{\text{L}}} { }c}}{{\left( {1 + K_{{\text{L}}} { }c} \right)}} \approx \frac{{{\Gamma }_{{{\text{max}}}}^{2} }}{{D{ }c^{2} }}.$$

In Eq. (2), the latter approximation holds true in the limit when the concentration is above a characteristic Langmuir concentration, $c \gg 1/K_{{\text{L}}}$, which is often several orders of magnitude below the CMC (see Theory S.2, SI, for full derivation). ${\Gamma }_{{{\text{max}}}}$, $K_{{\text{L}}}$, and $D$ are molecular properties that change with surfactant type (see Table S.2, SI, for property values). However, both ${\Gamma }_{{{\text{max}}}}$ and $D$ do not vary within an order of magnitude for many surfactants^20,41^; thus, given a particular surfactant, the diffusion time is almost solely dependent on the bulk concentration. From Eq. (2), $t_{{{\text{diff}}}}$ is inversely proportional to $c^{2} .$ Comparing the bubble timescale to the diffusion timescale as a ratio, when $t_{{\text{b}}} /t_{{{\text{diff}}}} < 1$, the diffusion of surfactants to the interface is slow and less surfactants will adsorb to the interfaces. According to Eq. (2), slow diffusion occurs at low concentrations. On the other hand, if $t_{{\text{b}}} /t_{{{\text{diff}}}} > 1$, diffusion is fast and more surfactants will adsorb to the interfaces, which corresponds to higher concentrations according to Eq. (2). Thus, the amount of adsorbed surfactant within a finite time window of $t_{{\text{b}}}$ is diffusion-transport-limited where the diffusion rate is set by the molar concentration.

**Figure 6:**
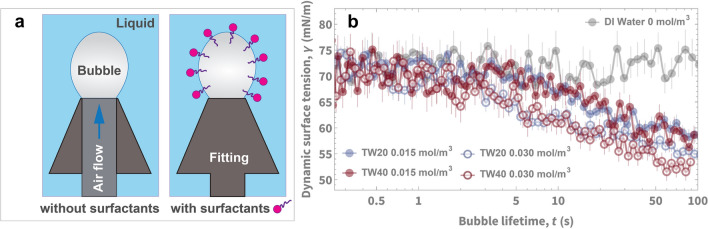
Surfactants with different CMCs and equilibrium surface tensions have similar dynamic adsorption behaviors at short time scales when at the same concentration. We performed (**a**) dynamic pendant bubble experiments to measure, using Young–Laplace fitting, (**b**) time-dependent surface tension of two surfactants: TW20 and TW40 at room temperature (25 °C) were used for testing at two different surfactant concentration amounts in DI water. The CMCs of TW20 and TW40 are 0.05 mol/m^3^ and 0.02 mol/m^3^, respectively. At 0.015 mol/m^3^ the equilibrium surface tensions are 47.66 ± 0.08 mN/m and 43.99 ± 0.09 mN/m, respectively. At 0.03 mol/m^3^ the equilibrium surface tensions are 44.22 ± 0.09 mN/m and 42.55 ± 0.01 mN/m, respectively. Despite the differences in equilibrium surface tensions, both surfactants had similar dynamic surface tensions, meaning that they have similar dynamic adsorption behaviors when at the same concentrations. The timescale of this similar dynamic adsorption (the diffusion timescale), decreases with increasing surfactant concentration.

To test whether a separate mechanism would affect our boiling data, such as a change on surface wettability, we considered long-term surface degradation change of the boiling copper tube over time. Past studies have found long-term oxidation effects^42^ and hydrocarbon adsorption effects^43^ can affect boiling. In our testing, we found that the dynamic contact angles did not change more than 4.5° before and after boiling (see Fig. S.5, SI). We have also included surface characterization of our boiling surface with FE-SEM images (Fig. S.6, SI). These results reveal no major surface wettability changes that will be the major cause affecting our experimental results. Aside from a permanent surface wettability change (long-term surface degradation), we considered another wettability aspect: surfactants adsorb to the liquid–vapor and solid–liquid interfaces, altering the actual wettability during boiling. This type of surfactant-mediated wetting effect is a challenging to characterize as bubble dynamics occur in timescales of milliseconds. In order to demonstrate that wettability is affected in a time-dependent manner, we conducted dynamic liquid–vapor surface tension measurements (see Fig. 6) on a bubble surrounded by surfactant solution in DI water at room temperature—a physical scenario that resembles the bubble formation in boiling. By characterizing the pendant shape of the bubble, we measure the surface tension as a function of time. We found that for two different types of surfactants (TW20 and TW40), their time-dependent surface tensions were very similar at the same concentrations (0.01 mol/m^3^ and 0.03 mol/m^3^). The results shown in Fig. 6 tell us that different surfactants do indeed adsorb to interfaces similarly at different concentrations, providing more substantiating evidence for why a universal peak occurs in HTC behavior in boiling. To further confirm the similarity in dynamic surface tensions at timescales faster than we can experimentally measure, we solved the Ward-Tordai equation to simulate dynamic surface tension at concentrations up to 100 mol/m^3^ in Fig. S.7, SI.

**Figure 7:**
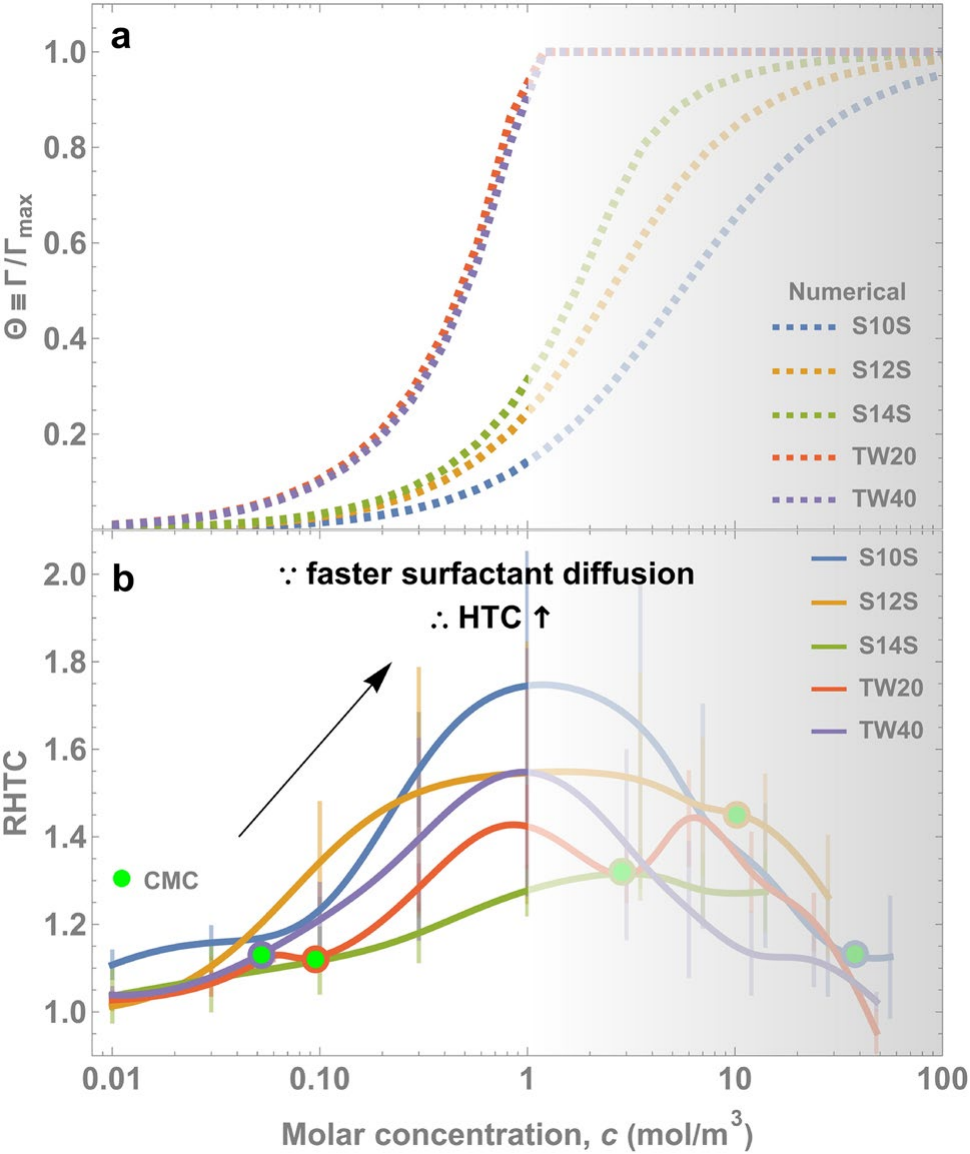
(**a**) We calculate the dynamic adsorption for a bubble lifetime of 20 ms (see Theory S.1, SI) using the Ward-Tordai equation^44^. The $\Gamma$ increases with concentration because of the faster diffusion rate as quantified by Eq. (2). All surfactants begin to increase in $\Gamma$ on the order of 0.1 mol/m^3^, which (**b**) coincides with the beginning of enhancement in RHTC.

To verify the effect of dynamic adsorption, we calculated the precise amount of adsorption within a time window and compared that to the amount of boiling enhancement. We solved the Ward-Tordai equation^44^ using an algorithm by Li et al.^45^ to calculate dynamic adsorption where surfactant properties were determined from our previous model^46^. Representing the dynamic adsorption nondimensionally as $\Theta \equiv\Gamma$/${\Gamma }_{\mathrm{max}}$ and using surfactant properties (see Theory S.3, SI), we found that adsorption indeed coincided with HTC enhancement below 1 mol/m^3^ (Fig. 7a) similarly for all surfactants. This unified enhancement behavior (Fig. 7b) can be explained by the fact that ${\Gamma }_{\mathrm{max}}$ and $D$ do not vary within an order of magnitude for many surfactants^20,41^. As a result, the ${t}_{\mathrm{diff}}$ dependence on molar concentration should be similar for many types of surfactants.

**Figure 8:**
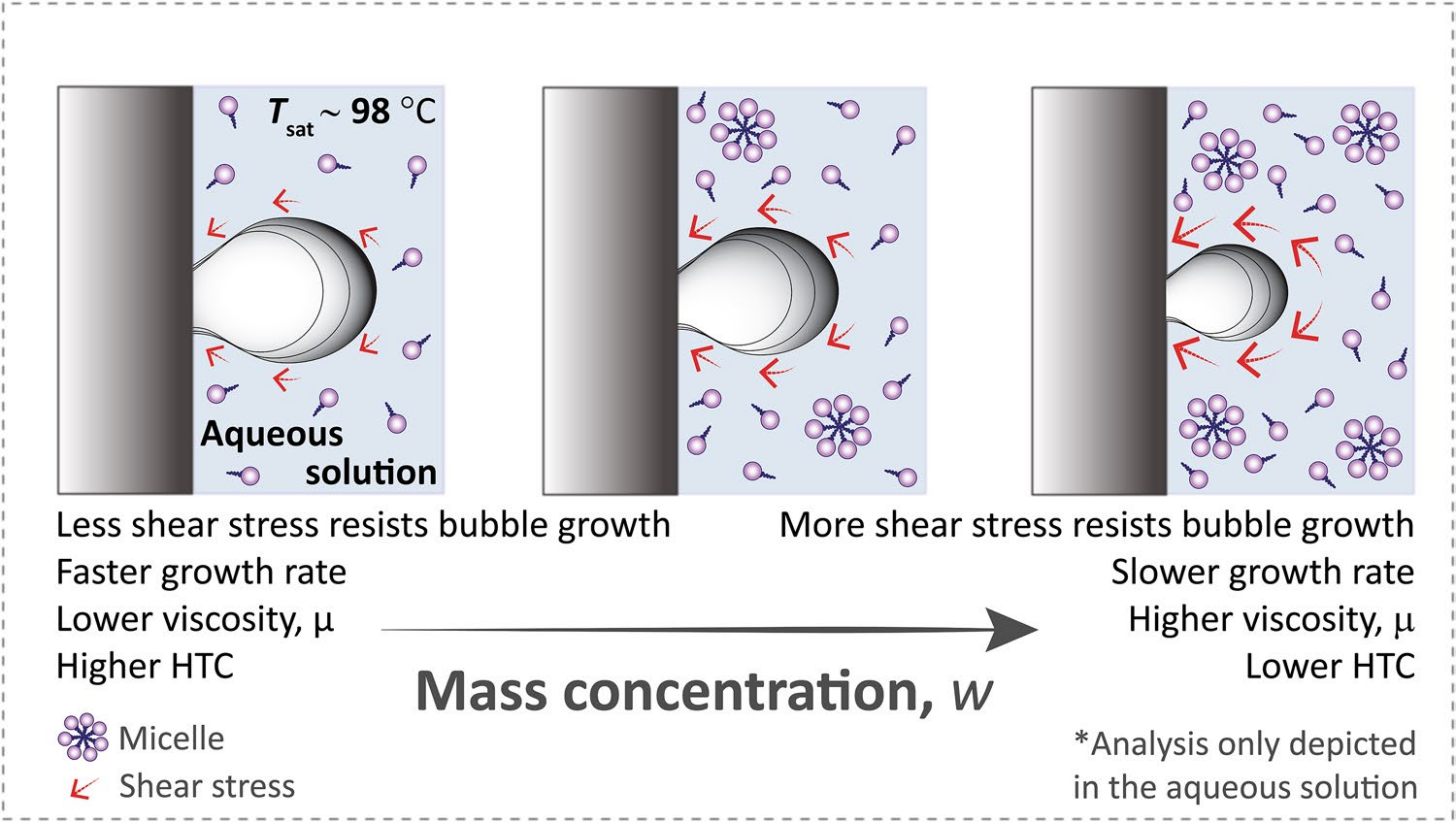
At higher surfactant concentration amounts, the HTC degrades due to increased viscosity resisting bubble growth.

This enhancement, however, does not continue forever. We hypothesize that this degradation is due to increased viscosity, $\mu$, (Fig. 8) since surfactants increase viscosity as concentration increases^47,48^ and boiling correlations indicate lower HTCs with higher viscosity^49,50^. Specifically, from the Rohsenow correlation^50^, the HTC is proportional to the inverse square of viscosity: HTC $\propto$ ${\mu }^{-2}$. We evaluated the viscosity of TW40 and S12S at various concentrations with a custom viscometer using the falling sphere method (see Methods and Fig. S.3, SI). We confirmed that the viscosity did increase above a certain concentration (Fig. S.4, SI) in agreement with previous study^48^.

**Figure 9:**
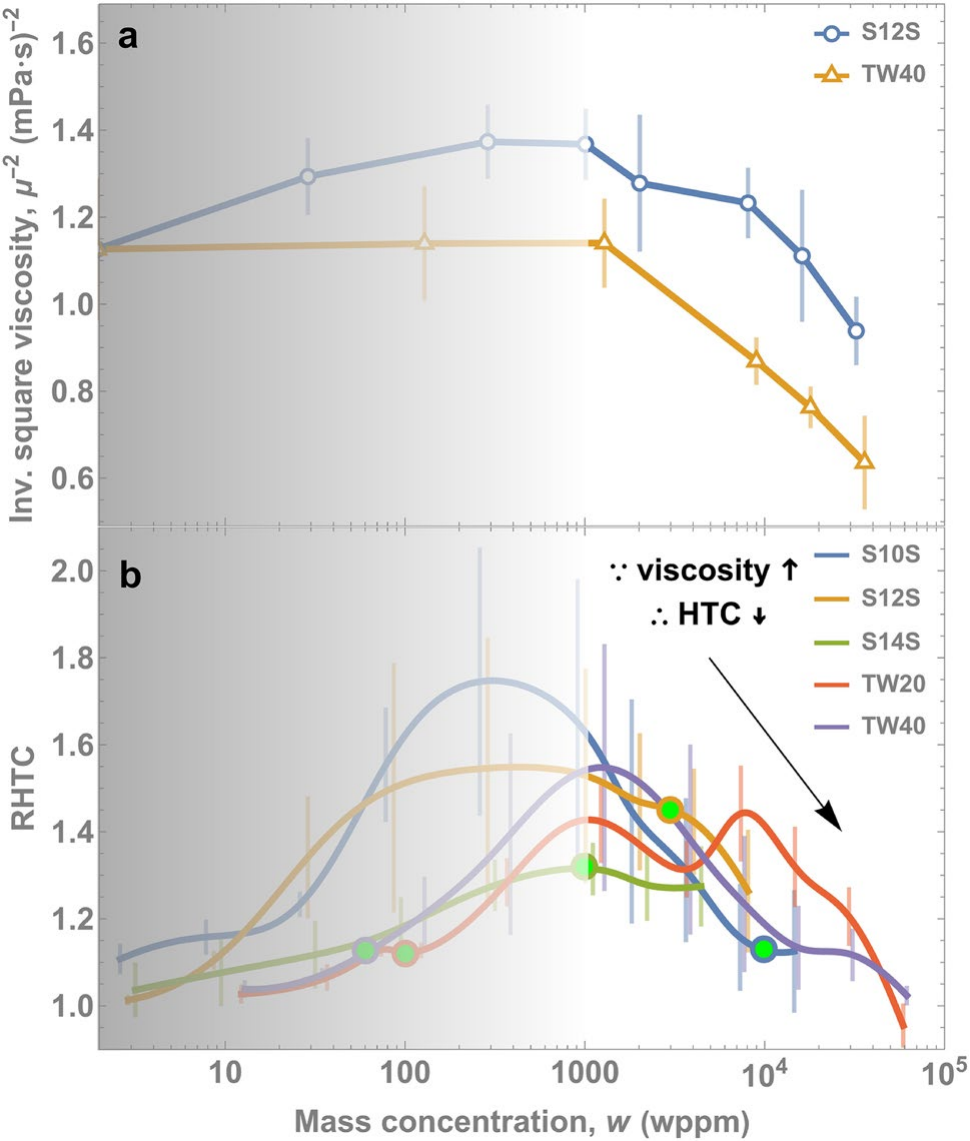
(**a**) The viscosity increases at high mass concentration values, which according to the Rohsenow correlation^50^, HTC ∝ μ^-2^. Indeed, (**b**) there a corresponding drop in RHTC with the increase in viscosity. Error bars in viscosity (one standard deviation in data deviations from measurements of the drop velocity and the ball bearing diameter and density). For more information about the propagation of errors, see Methods.

To illustrate the effect on HTC, we plot ${\mu }^{-2}$ in Fig. 9a as this should be proportional to the HTC. Indeed, the drop off in RHTC in Fig. 8b is concomitant with the drop in ${\mu }^{-2}$ in Fig. 9a, supporting our hypothesis that increased viscosity induces a heat transfer degradation.

From this point, we have established a link between dynamic adsorption and RHTC enhancement as well as a link between viscosity and RHTC degradation through the qualitative similarity between Figs. 7a, b and 9a, b, respectively. To further confirm these links quantitatively, we postulate a form of the proportionality in the Rohsenow boiling correlation that is affected by the surfactant concentration where

3 $${\text{HTC}} \propto \frac{{1 + a\left( {{\Gamma }\left( {\text{c}} \right)/{\Gamma }_{{{\text{max}}}} } \right)^{b} }}{{\mu \left( c \right)^{2} }}{\Delta }T^{2} .$$

**Figure 10:**
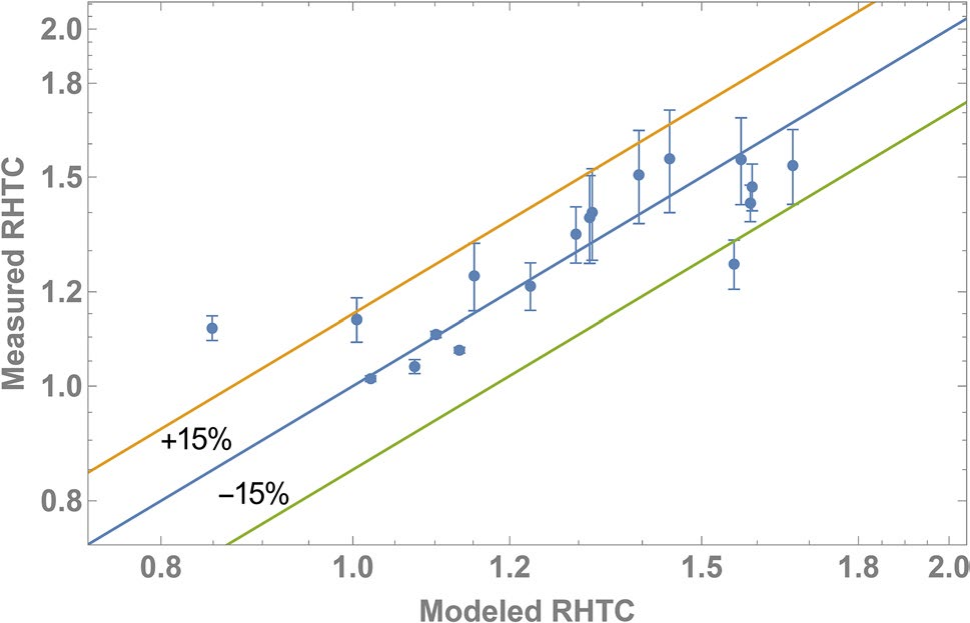
There is good agreement between the measured RTHC (Figs. 6b and 8b) and the modeled RHTC using Eq. (3) for all tested surfactants and concentrations. Our modeled RTHC incorporates the effects of HTC enhancement via adsorption and HTC degradation via viscosity increase.

Here, $a$ and $b$ are positive parameters that can be fit to our data. Equation (3) has appropriate limits where for pure water, ${\Gamma }\left( {\text{c}} \right)/{\Gamma }_{{{\text{max}}}} = 0$; thus, the proportionality reduces to the Rohsenow result of ${\text{HTC}} \propto \frac{1}{{\mu^{2} }}{\Delta }T^{2}$. The nondimensional adsorption, ${\Gamma }\left( {\text{c}} \right)/{\Gamma }_{{{\text{max}}}}$, can be solved using the Ward-Tordai equation. Both ${\Gamma }\left( {\text{c}} \right)/{\Gamma }_{{{\text{max}}}}$ and $\mu \left( c \right)$ are functions of concentration; $\mu \left( c \right)$ was experimentally determined for TW40 and S12S. As such, fitting the proportionality to the RHTC data, we were able to find a least-squares fit for $a$ and $b$ as 0.77 and 0.18, respectively. As shown in Fig. 10, we are able to model the RHTC using Eq. (3) to within + /− 15% error. This good, quantitative agreement provides a strong verification of the dual role that surfactants play in boiling: enhancers via adsorption and degraders via viscosity.

## Discussion

We have shown that, counter to conventional wisdom, surfactants have a fairly uniform concentration range of optimal enhancement in boiling. This concentration range is independent of CMC—which is an equilibrium property. Rather, HTC enhancement occurs due to a bubble-lifetime-limited adsorption of surfactants, which increases with concentration. This time-limited adsorption is controlled by surfactant properties that describe dynamic mass transport: diffusion coefficient and the maximum surface concentration of the surfactant. The dynamic surface tension measurements depicted in Fig. 6, show that different surfactants do indeed adsorb to interfaces similarly at similar concentrations, providing more substantiating evidence for why a universal peak occurs in HTC behavior in boiling. At very high concentrations, the HTC degrades due to an increase in viscosity. The increase of liquid viscosity with high surfactant concentration is concomitant with a decrease in heat transfer (Fig. 9). This suggests that a more viscous bulk solution resists bubble growth, resulting in smaller bubbles and slower growth rates as illustrated in Fig. 8, ultimately resulting in less heat transfer since boiling heat transfer is directly proportional to vapor generation rate.

We quantify the combined effect of adsorption and viscosity through a proportionality that can be added to the Rohsenow correlation. This proportionality can be expressed as a power law of $\frac{{1 + 0.77\left( {{\Gamma }\left( {\text{c}} \right)/{\Gamma }_{{{\text{max}}}} } \right)^{0.18} }}{{\mu \left( c \right)^{2} }}$, indicating the enhancing action of adsorption in the numerator and the degrading action of viscosity in the denominator. Our work resolves an important question of how surfactants can both enhance or degrade heat transfer through a mechanistic and molecular perspective. Specifically, we emphasize that surfactant-enhanced boiling is a dynamic process rather than an equilibrium process. The insights gained from this work will inform specific strategies to incorporate surfactant-enhanced boiling in a large array of two-phase heat transfer processes.

## Methods

### Pool boiling experiments

**Figure 11:**
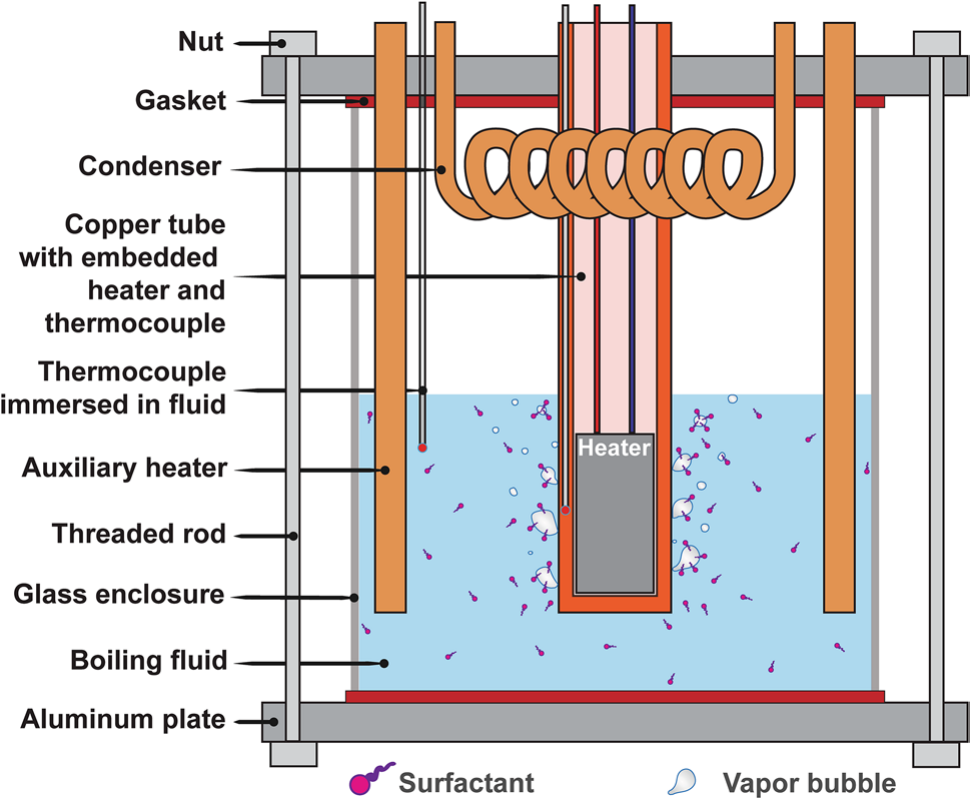
Schematic of the boiling setup. Two auxiliary heaters boil 300 ml of fluid to maintain a saturated temperature of ≈98 ℃ (saturation conditions in Las Vegas, NV, USA). Prior to the commencement of the boiling experiments, we degas the fluid for about 15 min. For every batch of experiments, we conduct DI water tests and gradually increase the concentration of the aqueous solution to run the tests at various surfactant concentrations.

Boiling experiments are performed at atmospheric pressure (≈1 atm) with DI water or aqueous solutions of surfactants in a custom boiling chamber (see Fig. 11 and Fig. S.1, SI). For each experiment, the heat flux was varied from 0 to 50 W/cm^2^. For each aqueous solution, we evaluate 10–12 molar concentrations, *c*, where each concentration is tested three times (including the boiling fluid with no surfactant; 0 mol/m^3^ representing deionized water), amounting to over 150 boiling experiments overall. The heating surface is carefully polished with a fine grit sandpaper for a mirror-like surface finish (for a detailed view of the heating surface, see Fig. S.5, SI). All the copper parts submerged in the boiling fluid are cleaned and rinsed with DI water and alcohol. The inside of the boiling chamber follows the same cleaning procedures. To remove organic/inorganic and microbial surface contaminants on the glass enclosure, we apply a plasma treatment for about 15–30 s. We evaluated the dynamic contact angles of the heating surface, before the commencing the experiments (pre-boiling) and after testing (post-boiling). Figure S.6 reveals there were not significant wettability changes on the surface of the copper tube.

### Viscosity measurements

We recorded videos at a set frame rate of 120 FPS using a camera positioned far away from the viscometer (Fig. S.3a). The viscometer consists of 3D printed resin parts, a rectangular borosilicate glass enclosure, a light source on the back of the glass container to provide enough illumination, a thermocouple for temperature measures of the fluid, and a magnetic stir bar to mix the aqueous solution of surfactant in the fluid (Fig. S.3b). We use a micropipette to add/remove aqueous solution through a small orifice on the top plate to reach the desired concentration value. For viscosity measurements, we use (Fig. S.3b) the falling sphere (stainless steel bearing balls) method. Experiments are conducted at a room temperature of 24–25 °C. We use an image processing technique to estimate the velocity of the ball bearing in a set region (pink rectangle) using Wolfram Mathematica. A custom 3D printed ruler is used for pixel calibration and estimate the distance traveled by the ball bearing in the set region. Then, we estimate the viscosity of the fluid based on the recorded velocity values.

### Uncertainty and propagation of error calculations

Error bars in Figs. 7b and 9b represent one standard deviation in relative HTC (RHTC) variation due to averaging of the heat flux, $q^{\prime\prime}$,

4 $$\Delta {\text{RHTC}}^{2} = \left( {\frac{\partial f}{{\partial q^{\prime\prime}}}} \right)^{2} {\Delta }q^{{\prime\prime}{2}} .$$

Similarly, uncertainty bars in Fig. 9a represent one standard deviation in viscosity, $\mu$, measurements accounting the errors of the drop velocity, $\nu$, and the ball bearing (diameter,$d$, and density, $\rho$)

5 $$\Delta {\upmu }^{2} = \left( {\frac{\partial f}{{\partial \nu }}} \right)^{2} {\Delta }\nu^{2} + \left( {\frac{\partial f}{{\partial d}}} \right)^{2} {\Delta }d^{2} + \left( {\frac{\partial f}{{\partial \rho }}} \right)^{2} {\Delta }\rho^{2} .$$

We performed three sets of experiments for every concentration amount totaling over 150 boiling experiments in this work. We have assessed the run-to-run repeatability of our experiments by considering the error across these three experiments for each concentration. We found that the variation in temperature was 0.14 ± 0.07 °C and the variation in heat flux was 0.57 ± 0.06 W/cm^2^; thus, boiling experiments were highly repeatable from run to run. We have also assessed the repeatability of our experiments as they changed from surfactant to surfactant. Before a series of experiments at various concentrations for a particular surfactant was run, we renewed the surface through polishing and cleaning as detailed above. Contact angles did not change significantly as a result of this renewal process (Fig. S.5). Pure DI water was tested in each case for repeatability and we found that the heat flux was within 2.8 ± 1.6 W/cm^2^. Thus, there is some repeatability error between series of experiments; however, this error in heat flux compared to the heat flux range that we test.

## Supplementary Information

Supplementary Information 1.

Supplementary Information 2.

## Data Availability

The data that support the findings of this study are available within the article, its supplementary information, which includes a JSON file of all data used in this work.
