# Supplementary material for: How dynamic adsorption controls surfactant-enhanced boiling
Source: Sci Rep. 2022 Oct 28;12:18170. doi: 10.1038/s41598-022-21313-1 (PMC9616907; doi:10.1038/s41598-022-21313-1)
Supplement: Supplementary file 1 — Supplementary Information 1. [file 41598_2022_21313_MOESM1_ESM.pdf]

SUPPLEMENTARY INFORMATION (SI)

How dynamic adsorption controls surfactant-enhanced boiling

Mario R. Mata, Brandon Ortiz, Dhruv Luhar, Vesper Evereux, and H. Jeremy Cho*

Department of Mechanical Engineering, University of Nevada, Las Vegas, Las Vegas, NV 89154, USA

The following supplementary information contains figures, tables, theories, and references that provide more details of the work presented in the main article.

SUPPLEMENTARY FIGURES

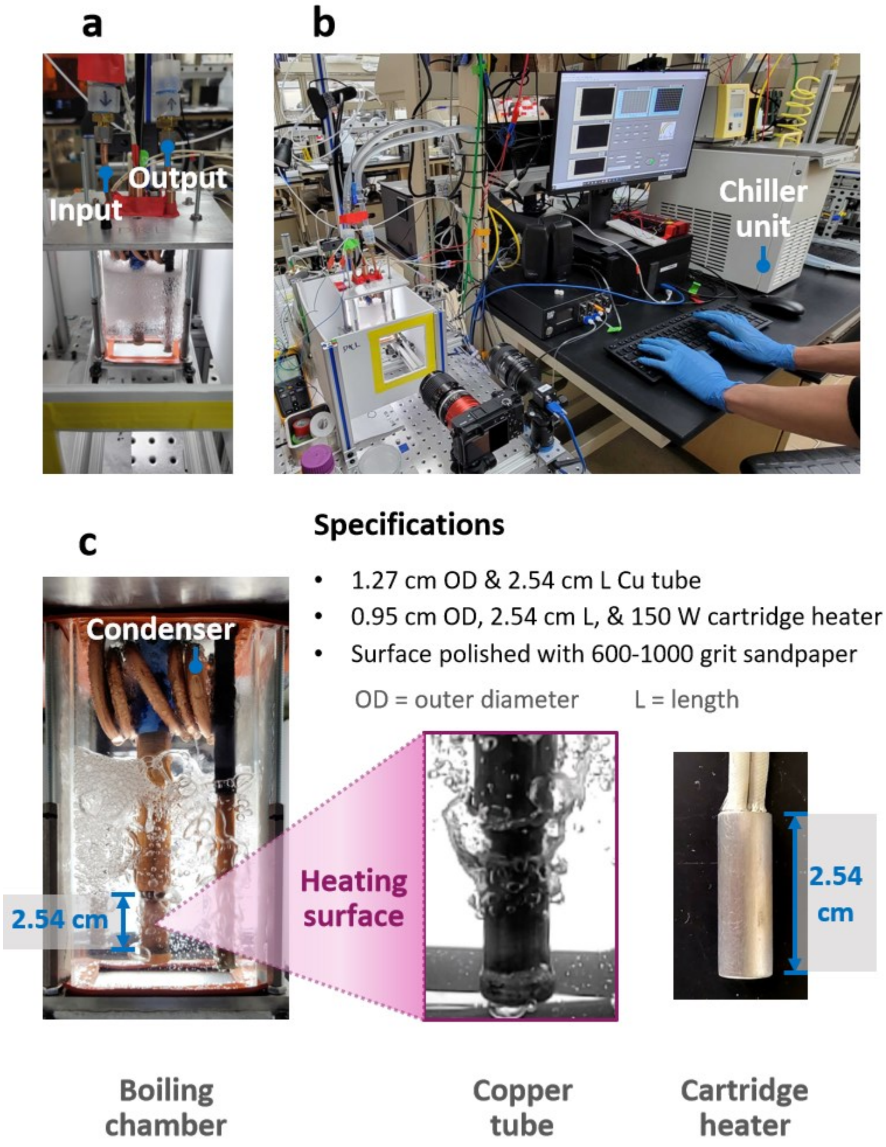

FIG. S.1. Boiling setup and specifications. The condenser is connected to (a) two lines that are directly connected to (b) a chiller unit, an input and an output line. These two lines make a loop which is circulating ethylene glycol at a set temperature of 15 ℃. (c) The heating element is a vertically oriented copper tube with an embedded cartridge heater.

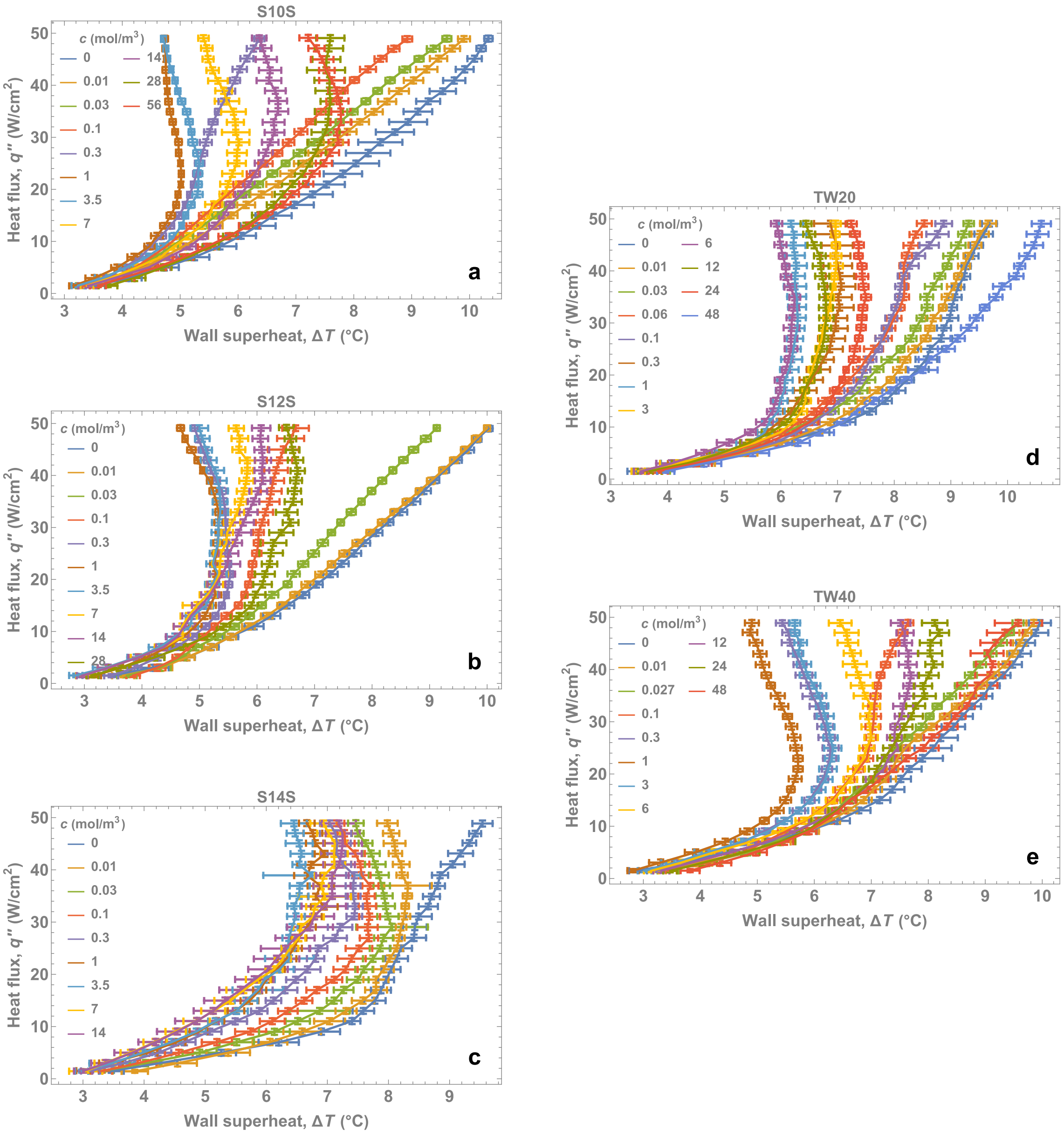

FIG. S.2. The heat flux as a function of the wall superheat. To ensure all bubble nucleation sites were activated on the heating surface[1], we record heat flux-averaged descending boiling curves (heat flux range: 50-1 W/cm^2 & sample range: 1 sample every 2 W/cm^2) under saturation conditions (fluid temperature ~ 98 ℃ and atmospheric pressure ~ 1 atm). The boiling experiments reveal that regardless of the type of the surfactant, plots (a), (b), (c), (d), and (e) show a decrease in the wall superheat as the molar concentration, c, increases. This trend continues up to a concentration range of c ~ 1-3.5 mol/m^3, which enhances the HTC. Above this range c > 1-3.5 mol/m^3, the wall superheat ramps back up to higher temperature values exacerbating the HTC.

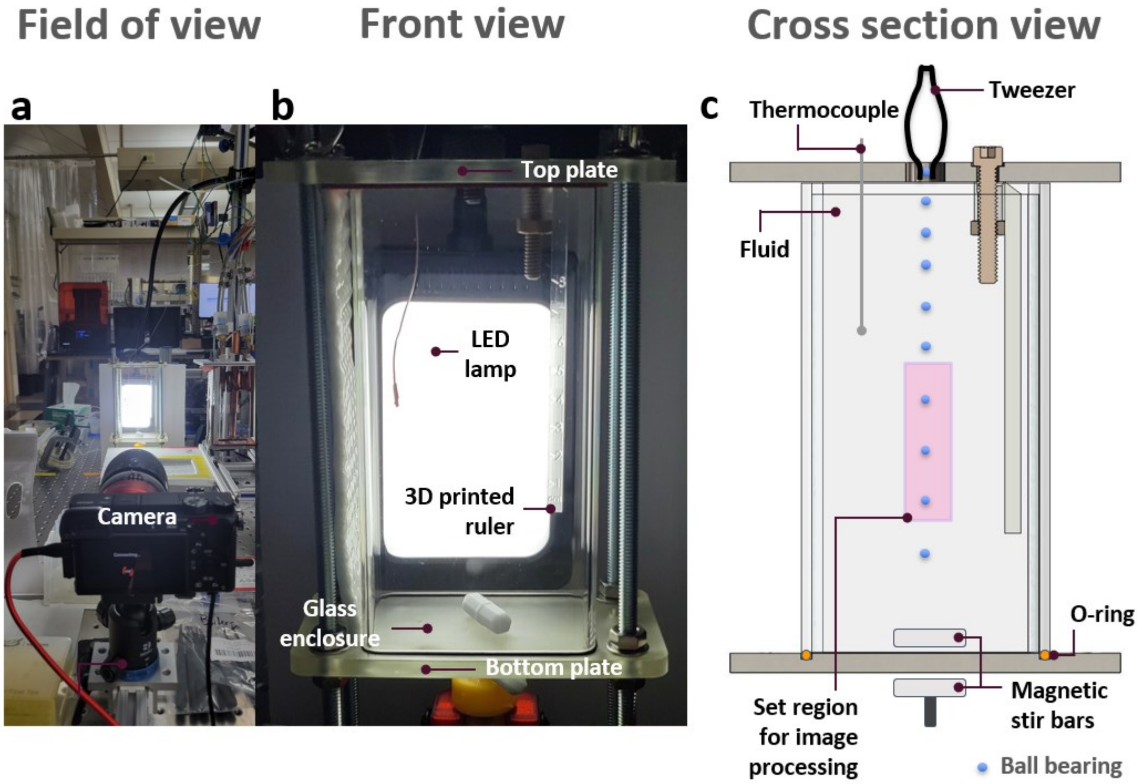

FIG. S.3. Custom viscometer setup. We recorded videos at a set frame rate of 120 FPS using (a) a camera positioned far away from the viscometer. (b) The viscometer consists of 3D printed resin parts, a rectangular borosilicate glass enclosure, a light source on the back of the glass container to provide enough illumination, a thermocouple for temperature measures of the fluid, and a magnetic stir bar to mix the aqueous solution of surfactant in the fluid. We use a micropipette to add/remove aqueous solution through a small orifice on the top plate to reach the desired concentration value. For viscosity measurements, we use (c) the falling sphere (stainless steel bearing balls) method. Experiments are conducted at a room temperature of $24{\sim}25$ ℃. We use an image processing technique to estimate the velocity of the ball bearing in a set region (pink rectangle) using Wolfram Mathematica. A custom 3D printed ruler is used for pixel calibration and estimate the distance traveled by the ball bearing in the set region. Then, we estimate the viscosity of the fluid based on the recorded velocity values.

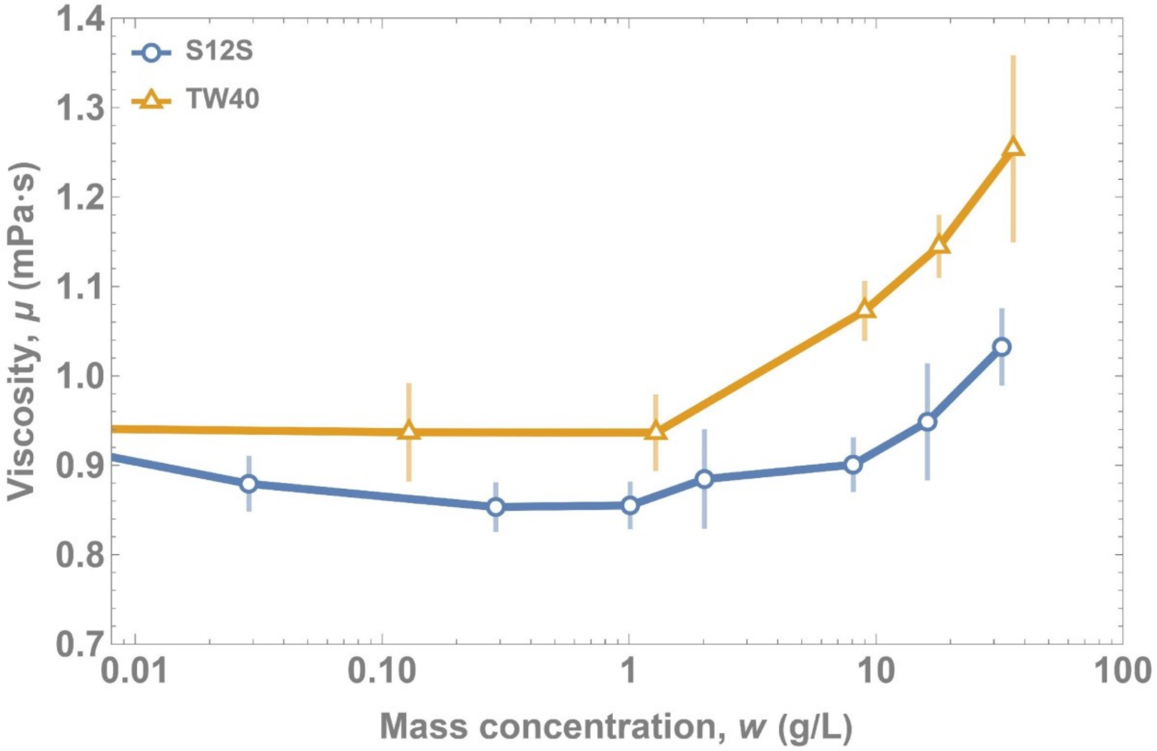

FIG. S.4. Results of viscosity tests. Experiment tests conducted at room temperature conditions (24~25℃). At a relatively low molar concentration, both surfactants, S12S and TW40, show minor changes in the viscosity. As we increase the mass concentration of surfactant, both aqueous solutions become more viscous, with an apparent increase in viscosity when $c > 1$ mol/m^3. A small dip in viscosity from 0.01 to 1 g/L is likely due to measurement error and drifting temperature conditions in the laboratory.

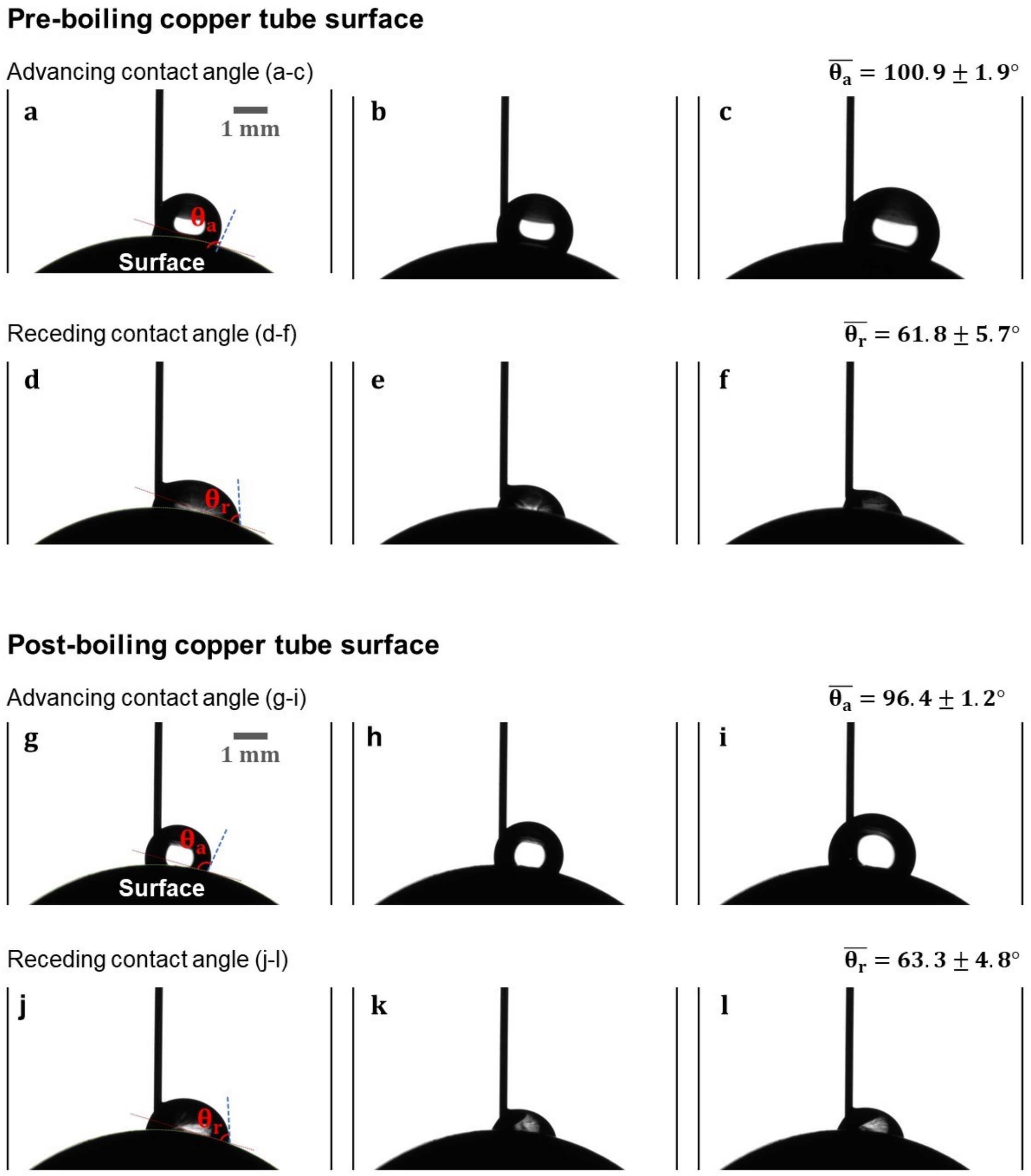

FIG. S.5. Dynamic contact angle measurement. The averaged dynamic contact angles before and after testing show that the experimental sample (copper tube) did not exhibit a significant change in wettability. These contact angle tests of DI water droplets were conducted at room temperature conditions (≈23 °C) using a custom-built goniometer.

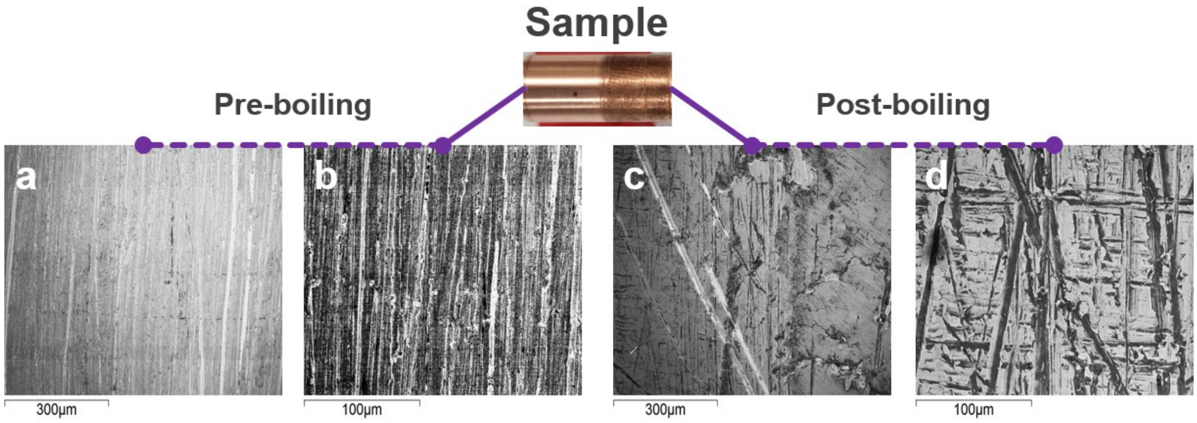

FIG. S.6. Field emission scanning electron microscopy (FE-SEM) images. Two randomly locations, one on each side, were selected to qualitative characterize the surface of the experimental sample (copper tube) used in the boiling experiments. On the left-hand side of the sample, (a & b) show the morphology before testing (pre-boiling). On the right-hand side, (c & d) show the sample after testing (post-boiling). The sample was polished with a 1000-grit sandpaper.

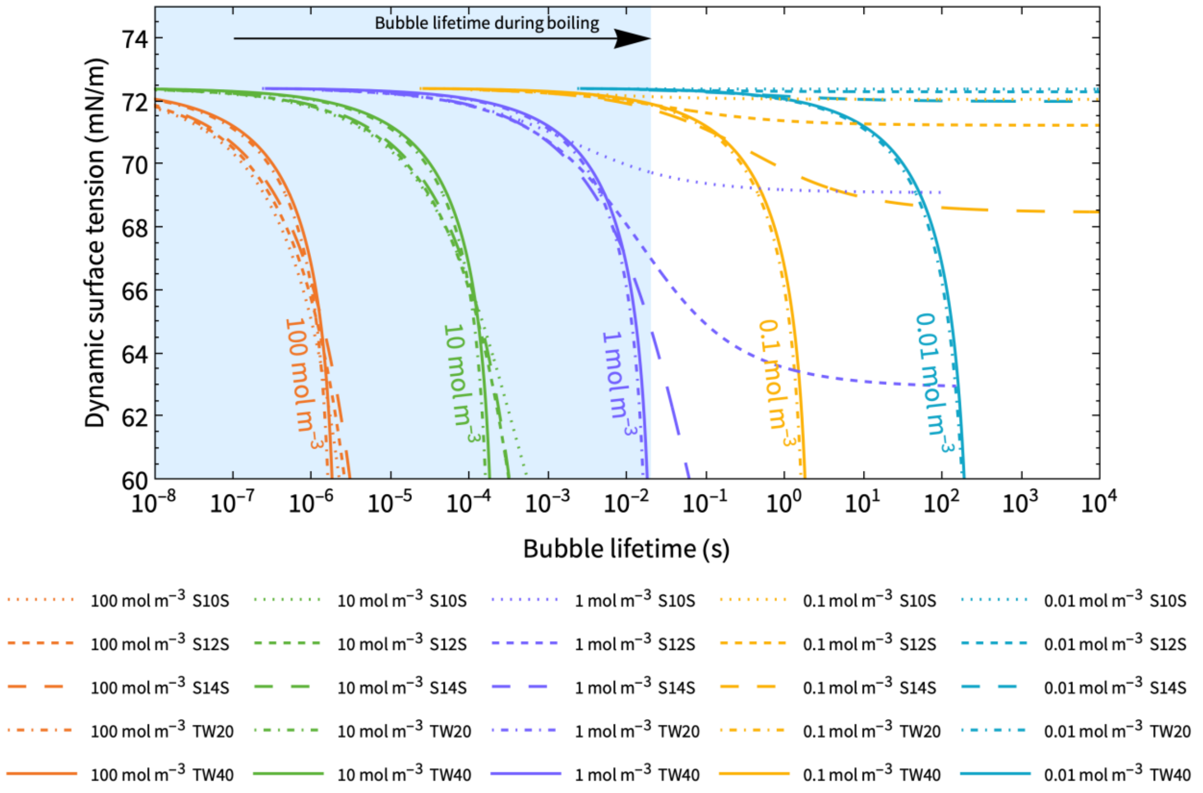

FIG. S.7. Modeled dynamic surface tension results show that surface tension drops similarly at short timescales below the bubble lifetime in boiling (20 ms) for all surfactants at the same concentration. As concentration increases, the timescale of surface tension drop decreases. Specifically, as the concentration is increased by 10x, the timescale decreases by 100x. This is in agreement with Eq. 2 of the main text where $t_{\mathrm{diff}} \propto c^{-2}$. Above 1 mol m^{-3}, all surfactants drop similarly within the boiling bubble lifetime of around 20 ms. Dynamic surface tension results were obtained by numerically solving the Ward-Tordai equation (see section S.3) with the data from Table S.2.

SUPPLEMENTARY TABLES

TABLE. S.1. Physico-chemical properties of surfactants

Surfactant	Abbreviation	Type	Molecular weight (g/mol)	Carbons groups in the tail	CMC (mol/m^3) [a]	
					At 25 °C	At 98 °C
Sodium decyl sulfate	S10S	Anionic	260.33	10	32.2	37.9
Sodium dodecyl sulfate	S12S	Anionic	288.38	12	7.4	10.2
Sodium tetradecyl sulfate	S14S	Anionic	316.44	14	1.8	2.9
Polysorbate 20	TW20	Nonionic	1,227.54	12	0.05	0.1
Polysorbate 40	TW40	Nonionic	1,283.63	16	0.02	0.05

[a] Critical micelle concentration (CMC) values for all the surfactants, except TW40, were calculated using PREDICT software by Zoeller and Blankschtein[2]. TW40 CMC values were obtained by data reported (at 25 °C) and extrapolated (at 98 °C) by Mohajerani and Noudeh[3].

TABLE. S.2. Dynamic adsorption properties of surfactants at room temperature (25 °C)

Surfactant	Γ_{max} (10^{-6} mol/m^2) [a]	K_{L} (m^3/mol) [a]	D (10^{-10} m^2/s) [b]
S10S	7.32394	0.20029	5.16383
S12S	8.04953	0.60952	4.43817
S14S	8.58987	2.03147	3.87403
TW20	2.02024	8,681.9	1.73323
TW40	2.02324	18,189.3	1.53725

[a] Values calculated from surface tension data for TW20 and TW40 reported by Din et al.[4]. [b] Values obtained using a method for calculating the diffusion constant presented by Cho et al.[5].

THEORY

S.1. Bubble lifetime

Referring to Cole's work, equation (16)[6], we calculate the bubble departure diameter during nucleate boiling (with a wall superheat, ΔT, in the range of $6-7$ °C; see our boiling curves in Fig. S.2). The departure diameter, D_d, is proportional to some function of the Jakob Number, N_Ja, which is

$$D_\mathrm{d} \sim \left[\frac{\sigma}{g\,(\rho_\mathrm{l} - \rho_\mathrm{v})}\right]^{1/2} N_\mathrm{Ja}, \tag{S.1.1}$$

where

$$N_\mathrm{Ja} = \frac{\rho_\mathrm{l}}{\rho_\mathrm{v}}\, c_\mathrm{p} \frac{\Delta T}{h_\mathrm{fg}}. \tag{S.1.2}$$

Here, σ is the surface tension, g is the gravitational acceleration, ρ_l and ρ_v are the densities of the liquid and vapor, respectively, c_p is the specific heat at constant pressure, and h_fg is the specific latent heat of vaporization.

Solving for the bubble departure diameter with typical properties in boiling water, we find $D_\mathrm{d} = 1.95 \pm 0.21$ mm. Then, we refer to Cole's equation (25)[6] to calculate the bubble frequency, f_s:

$$f_\mathrm{s}\, D_\mathrm{d} \sim \left[g\,\frac{(\rho_\mathrm{l} - \rho_\mathrm{v})}{{\rho_\mathrm{l}}^2}\,\sigma\right]^{1/4}. \tag{S.1.3}$$

We solve for the bubble frequency and get $f_\mathrm{s} = 48.5 \pm 3.7$ Hz. Taking the inverse of f_s (where 1 Hz = 1 cycle/s), we get a bubble cycle period (bubble lifetime, t_b) of 20.6 ± 2.2 ms.

S.2. Characteristic timescale of diffusion

Most surfactants follow[7] the non-linear Langmuir isotherm, defined as

$$\Gamma_{\mathrm{eq}} = \Gamma_{\mathrm{max}} \frac{K_{\mathrm{L}}\, c}{1 + K_{\mathrm{L}}\, c} \tag{S.2.1}$$

where Γ_{eq} is the equilibrium surface concentration, Γ_{max} is the maximum surface concentration (a theoretical limit[7]), K_{L} is the Langmuir equilibrium adsorption constant, and c is bulk concentration.

From Ferri and Stebe's work[8], the characteristic time scale is equivalent to a characteristic length scale squared divided by the diffusion coefficient—as with any diffusion phenomenon:

$$t_{\mathrm{diff}} = \frac{h^2}{D}. \tag{S.2.2}$$

Here, the characteristic length scale h, is the adsorption depth depleted by surfactant adsorption, and D is the diffusivity of surfactant in the solution. Most surfactants have a $D \sim 10^{-10}$ m^2/s, as shown in Table S.2.

We can solve for the characteristic length by considering that when dissolved surfactants beneath a surface are adsorbed to the surface, there is a length scale of depletion, h. Initially, at $t = 0$, the surface has no adsorbed surfactant. As surfactants adsorb toward an equilibrium amount of $\Gamma_{\mathrm{eq}}\, \delta A$, this amount must equal what was originally beneath the surface, $c\, h\, \delta A$. Thus, $\Gamma_{\mathrm{eq}}\, \delta A = c\, h\, \delta A$. Solving for h,

$$h = \frac{\Gamma_{\mathrm{eq}}}{c}. \tag{S.2.3}$$

From here, we substitute equation (S.2.3) into equation (S2.2) to get the timescale equation in terms of Γ_{eq}

$$t_{\text{diff}} = \frac{{\Gamma_{\text{eq}}}^2}{c^{\,2}\,D}. \tag{S.2.4}$$

Then, we resubstitute equation (S.2.4) into the original Langmuir equation (S.2.1) to find the corresponding characteristic timescale of diffusion, which is

$$\text{t}_{\text{diff}} = \frac{{\Gamma_{\text{max}}}^2\left(\frac{K_{\text{L}}\,c}{1+K_{\text{L}}\,c}\right)}{c^{\,2}\,D} \approx \frac{{\Gamma_{\text{max}}}^2}{c^{\,2}\,D}. \tag{S.2.5}$$

In equation (S.2.5), we apply the simplifying approximation in the limit of high concentration, $c \gg 1/K_{\text{L}}$, and this is due to the fact that c is larger compared to $1/K_{\text{L}}$, which occurs several orders of magnitude below the critical micelle concentration (CMC) for any type of surfactant. Very high $K_{\text{L}}\,c$, represents a very high relative concentration, which is close to the CMC, CMC $\gg 1/K_{\text{L}}$. In other words, the product of $K_{\text{L}}\,c$ tell us a respective amount of (how high or low) the bulk concentration is compared to the relative concentration of $1/K_{\text{L}}$.

The equation of state[8], equation (S.2.6), tell us how much surface tension, γ, drop is associated with a particular surface concentration, Γ. Thus, when $c = 1/K_{\text{L}}$, the interface is said to be at 50% its capacity ($0.5 = \Gamma/\Gamma_{\text{max}}$), we should expect a small drop in surface tension (a few mN/m). Similarly, when $c \ll 1/K_{\text{L}}$, we get $K_{\text{L}}\,c \ll 1$ where the surface tension would be very near the value of pure water.

$$\gamma = \gamma_O + R\,T\,\Gamma_{\text{max}}\,\ln\left(1 - \frac{\Gamma}{\Gamma_{\text{max}}}\right) \tag{S.2.6}$$

where γ_O is the surface tension of the surfactant-free interface, and $R\,T$ is the product of the ideal gas constant and the temperature of the fluid. Solving for γ with dynamic adsorption properties of TW40 in water at room temperature ($23 - 25\,℃$), we get a drop in surface tension of 3.42 ± 0.02 mN/m.

S.3. Dimensionless Ward-Tordai equation

In this work, we solve the classic Ward-Tordai equation[9]

$$\Gamma(t) = \frac{2\,c\,\sqrt{D\,t}}{\sqrt{\pi}} - \frac{\sqrt{D}}{\sqrt{\pi}}\int_0^t \frac{c_0(\tau)}{\sqrt{t-\tau}}d\tau. \tag{S.3.1}$$

Here, τ is a dummy variable and c_0 is the subsurface concentration. The Ward-Tordai equation is a solution to the 1D, transient diffusion problem of surfactants migrating to a fresh interface.

From here, we define some convenient dimensionless variables. We nondimensionalize the subsurface concentration, c_0, with respect to the bulk concentration, c

$$C_0(t) \equiv \frac{c_0(t)}{c} = C(t,0). \tag{S.3.2}$$

Next, we nondimensionalize the surface concentration, Γ, with respect to the maximum surface concentration, $\Gamma_{\max}$

$$\Theta \equiv \frac{\Gamma}{\Gamma_{\max}}. \tag{S.3.3}$$

Lastly, we nondimensionalize time as

$$T \equiv \frac{t\,D\,c^2}{{\Gamma_{\max}}^2}. \tag{S.3.4}$$

Now, we divide equation (S.3.1) by $\Gamma_{\max}$

$$\frac{\Gamma(t)}{\Gamma_{\max}} = \left(\frac{2\,c}{\Gamma_{\max}}\frac{\sqrt{D\,t}}{\sqrt{\pi}}\right) - \frac{1}{\Gamma_{\max}}\frac{\sqrt{D}}{\sqrt{\pi}}\int_0^t \frac{c_0(\tau)}{\sqrt{t-\tau}}d\tau,$$

which then we get

$$\frac{\Gamma(t)}{\Gamma_{\max}} = \left(2\frac{\sqrt{\frac{t\,D\,c^2}{\Gamma_{\max}{}^2}}}{\sqrt{\pi}} \right) - \frac{1}{\sqrt{\pi}} \int_0^{\frac{t\,D\,c^2}{\Gamma_{\max}{}^2}} \frac{C_0(T')}{\sqrt{\left(\frac{t\,D\,c^2}{\Gamma_{\max}{}^2} - T'\right)}} dT'.$$

Hence, the dimensionless Ward-Tordai equation is

$$\Theta(T) = \frac{2\sqrt{T}}{\sqrt{\pi}} - \frac{1}{\sqrt{\pi}} \int_0^{T} \frac{C_0(T')}{\sqrt{T - T'}} dT'. \tag{S.3.5}$$

When C_0 is very low at short times, the integral term of equation (S.3.5) is approximately zero. Thus, the dynamic adsorption is approximated as

$$\Theta(T) = \frac{2\sqrt{T}}{\sqrt{\pi}}. \tag{S.3.6}$$

Equation (S.3.6) is used to calculate the dynamic adsorption of the five different surfactants shown in Fig. 7a. We use a numerical scheme to integrate equation S.3.5 developed by Li[10]. We replicated this algorithm in Mathematica with the following code.

Code:

```
getndwtresults[θvsKLc0data_, KLc∞_, startT_, endT_, stepsPerDecade_] :=
 Module[{T, θjumpdata, θcut1, θcut2, endtime, gh, C0, gterm, Kterm, θrootsolver, startingIntegrationIndex, times}, endtime = endT;
  θjumpdata = MaximalBy[Table[{i, θvsKLc0data[[i, 2]], θvsKLc0data[[i, 2]] - θvsKLc0data[[i - 1, 2]]}, {i, 2, Length[θvsKLc0data]}], Last];
  θcut1 = θvsKLc0data[[θjumpdata[[1, 1]] - 1, 2]];
  θcut2 = θjumpdata[[1, 2]];
  C0 = Interpolation[Append[({#1[[2]], #1[[1]]/KLc∞} &) /@ θvsKLc0data, {0, 0}], InterpolationOrder → 1];
  gterm[T_] := (2 Sqrt[T])/Sqrt[π];
  Kterm[T_, Tp_, θ_] := -(Sqrt[1/π] C0[θ])/Sqrt[T - Tp];
  θrootsolver[time_, θBound1_, θBound2_, h_] := Module[{tn, dx, xmid, rtb, f, fmid, k, gb1, gb2}, tn = time;
    gb1 /. FindRoot[gb1 - (h Kterm[tn, tn - 0.5` h, gb1] + θBound2), {gb1, θcut1, 0, Max[θvsKLc0data[[1 ;; -1, 2]]]}]];
  Module[{NN, totalTime, h, θs, n, sum, j, x, sums, logsteptimes, inittimes, diffinittimes, goodposinittimes, goodinittimes},
   totalTime = endtime;
   inittimes = (N[10^#1] &) /@ Subdivide[Log10[startT], Log10[totalTime], Round[stepsPerDecade (Log10[totalTime] - Log10[startT])]];
   diffinittimes = Table[inittimes[[i]] - inittimes[[i - 1]], {i, 2, Length[inittimes]}];
   goodposinittimes = Flatten[Position[diffinittimes, x_ /; x > startT]] + 1;
   goodinittimes = inittimes[[goodposinittimes]];
   times = Prepend[If[Length[goodinittimes] > 0, Join[Table[(goodinittimes[[1]] i)/Ceiling[goodinittimes[[1]]/startT], {i, Ceiling[goodinittimes[[1]]/startT] - 1}], goodinittimes],
      Table[i, {i, startT, endtime, startT}]], 0];
   NN = Length[times] - 1;
   θs = Table[0, {i, 0, NN}];
   sums = Table[0, {i, 0, NN}];
   startingIntegrationIndex = 1;
   For[n = 1, n ≤ NN, n = n + 1, sum = 0;
    h = times[[n + 1]] - times[[n]];
    If[n - startingIntegrationIndex == 1, sum = Sum[(times[[j + 1]] - times[[j]]) Kterm[times[[n + 1]], times[[j + 1]], θs[[j + 1]]], {j, startingIntegrationIndex, n - 1}];,
     If[n - startingIntegrationIndex > 1,
      sum = Sum[1/2 (times[[j + 1]] - times[[j]]) (Kterm[times[[n + 1]], times[[j + 1]], θs[[j + 1]]] + Kterm[times[[n]], times[[j]], θs[[j]]]), {j, startingIntegrationIndex, n - 1}];]];
    x = gterm[times[[n + 1]]] + sum;
    θs[[n + 1]] = θrootsolver[times[[n + 1]], θs[[n]], x, h];];
   Table[{times[[i]], θs[[i]]}, {i, Length[times]}]]]
```

REFERENCES

1. McHale, J. P., Garimella, S. v., Fisher, T. S. & Powell, G. A. Pool boiling performance comparison of smooth and sintered copper surfaces with and without carbon nanotubes. *CTRC Research Publications* **15**, 133–150 (2011).
2. Zoeller, N. J. & Blankschtein, D. Development of user-friendly computer programs to predict solution properties of single and mixed surfactant systems. *Ind Eng Chem Res* **34**, 4150–4160 (2002).
3. Mohajeri, E. & Noudeh, G. D. Effect of temperature on the critical micelle concentration and micellization thermodynamic of nonionic surfactants: polyoxyethylene sorbitan fatty acid esters. *E-Journal of Chemistry* **9**, 2268–2274 (2012).
4. Ud-din, K., Khan, A. B. & Naqvi, A. Z. Mixed micellization and interfacial properties of nonionic surfactants with the phenothiazine drug promazine hydrochloride at 30 °C. *J Solution Chem* **41**, 1587–1599 (2012).
5. Cho, H. J., Sresht, V. & Wang, E. N. Predicting surface tensions of surfactant solutions from statistical mechanics. *Langmuir* **34**, 2386–2395 (2018).
6. Cole, R. Bubble frequencies and departure volumes at subatmospheric pressures. *AIChE Journal* **13**, 779–783 (1967).
7. Chang, C. H. & Franses, E. I. Adsorption dynamics of surfactants at the air/water interface: a critical review of mathematical models, data, and mechanisms. *Colloids Surf A Physicochem Eng Asp* **100**, 1–45 (1995).
8. Ferri, J. K. & Stebe, K. J. Which surfactants reduce surface tension faster? A scaling argument for diffusion-controlled adsorption. *Adv Colloid Interface Sci* **85**, 61–97 (2000).
9. Ward, A. F. H. & Tordai, L. Time-dependence of boundary tensions of solutions I. The role of diffusion in time-effects. *J Chem Phys* **14**, 453–461 (1946).
10. Li, X., Shaw, R., Evans, G. M. & Stevenson, P. A simple numerical solution to the Ward–Tordai equation for the adsorption of non-ionic surfactants. *Comput Chem Eng* **34**, 146–153 (2010).
